# Prevalences and associated risk factors of HCV/HIV co-infection and HCV mono-infection among injecting drug users in a methadone maintenance treatment program in Taipei, Taiwan

**DOI:** 10.1186/1471-2458-12-1066

**Published:** 2012-12-11

**Authors:** Yung-Feng Yen, Muh-Yong Yen, Lien-Wen Su, Lan-Huei Li, Peing Chuang, Xiao-Ru Jiang, Chung-Yeh Deng

**Affiliations:** 1 Section of Infectious Diseases, Taipei City Hospital, Taipei City Government, Taipei, Taiwan; 2 Department of Disease Control and Prevention, Taipei City Hospital, Taipei City Government, Taipei, Taiwan; 3 Division of Mental Health and Addiction Medicine, Taipei City Hospital, Taipei City Government, Taipei, Taiwan; 4 Institute of Hospital and Health Care Administration, National Yang-Ming University, 155, Section 2, Ni-Long Street, Taipei, Taiwan; 5 Institute of Public Health, National Yang-Ming University, Taipei, Taiwan

**Keywords:** HCV, HIV, Taiwan, Injection drug use, Methadone

## Abstract

**Background:**

Injecting drug users (IDUs) in Taiwan contributed significantly to an HIV/AIDS epidemic in 2005. In addition, studies that identified risk factors of HCV/HIV co-infection among IDUs were sparse. This study aimed to identify risk factors of HCV/HIV co-infection and HCV mono-infection, as compared with seronegativity, among injecting drug users (IDUs) at a large methadone maintenance treatment program (MMTP) in Taipei, Taiwan.

**Methods:**

Data from enrollment interviews and HCV and HIV testing completed by IDUs upon admission to the Taipei City Hospital MMTP from 2006–2010 were included in this cross-sectional analysis. HCV and HIV testing was repeated among re-enrollees whose HCV or HIV test results were negative at the preceding enrollment. Backward stepwise multinomial logistic regression was used to identify risk factors associated with HCV/HIV co-infection and HCV mono-infection.

**Results:**

Of the 1,447 IDUs enrolled, the prevalences of HCV/HIV co-infection, HCV mono-infection, and HIV mono-infection were 13.1%, 78.0%, and 0.4%, respectively. In backward stepwise multinomial regression analysis, after controlling for potential confounders, syringe sharing in the 6 months before MMTP enrollment was significantly positively associated with HCV/HIV co-infection (adjusted odds ratio [AOR]=27.72, 95% confidence interval [CI] 13.30–57.76). Incarceration was also significantly positively associated with HCV/HIV co-infection (AOR=2.01, 95% CI 1.71–2.37) and HCV mono-infection (AOR=1.77, 95% CI 1.52–2.06), whereas smoking amphetamine in the 6 months before MMTP enrollment was significantly inversely associated with HCV/HIV co-infection (AOR=0.44, 95% CI 0.25–0.76) and HCV mono-infection (AOR=0.49, 95% CI 0.32–0.75). HCV seroincidence was 45.25/100 person-years at risk (PYAR; 95% CI 24.74–75.92/100 PYAR) and HIV seroincidence was 0.53/100 PYAR (95% CI 0.06–1.91/100 PYAR) among re-enrolled IDUs who were HCV- or HIV-negative at the preceding enrollment.

**Conclusions:**

IDUs enrolled in Taipei MMTPs had very high prevalences of HCV/HIV co-infection and HCV mono-infection. Interventions such as expansion of syringe exchange programs and education regarding HCV/HIV prevention should be implemented for this high-risk group of drug users.

## Background

Injecting drug users (IDUs) are susceptible to many blood-borne infections. It is estimated that there are 15.9 million IDUs worldwide [1], among whom 62.9% were infected with hepatitis C virus (HCV) and 18.9% with human immunodeficiency virus (HIV) [1,2]. Many human behaviors, such as unsafe injection practices and unsafe sexual behaviors, are associated with HCV [3] and HIV [4,5] infections among IDUs. HCV and HIV contribute substantially to morbidity and mortality in this population [2,6]. Moreover, co-infection with HCV and HIV is not uncommon and has become a global public health problem, causing an increased rate of progression to cirrhosis, decompensated liver disease, hepatocellular carcinoma, and death [7,8]. Therefore, it is crucial to determine the prevalences of HCV/HIV co-infection and HCV and HIV mono-infection among IDUs.

In Taiwan, the problem of HCV and HIV infection among IDUs has received increasing attention. HCV prevalence appears to be very high among IDUs, with prevalences estimated at between 59.5% and 89.6% for IDUs [9-12] as compared with 4.4% among Taiwanese aged 20 years or older [13]. Additionally, HIV prevalence was estimated to be between 12.3% and 25.5% among IDUs, as compared with 0.08% among the general population [10,12,14]. Of even greater concern is that from 2003 to 2005 newly reported HIV-positive cases increased rapidly from 860 to 3381, and the percentage attributed to IDUs markedly increased from 2.1% to 72.4%. To curb this outbreak, Taiwan’s Centers for Disease Control (CDC) began a harm reduction program in 2006 that provides syringe exchange services at pharmacies and methadone clinics and offers methadone therapy to heroin addicts. Taiwan’s HIV epidemic has slowed since then—in 2011 the number of newly reported HIV cases decreased to 1963, and the percentage attributed to IDUs decreased to 6.4%. However, IDUs still accounted for 29.9% of all reported HIV cases in Taiwan at the end of 2011 [14].

The epidemiology of HCV and HIV mono-infection and HCV/HIV co-infection among IDUs has not been thoroughly studied, and no study has investigated risk factors associated with HCV/HIV co-infection in Taiwan. Only two studies—one in Vancouver [15] and another in southern China [16]—distinguished between HCV mono-infection and HCV infection among IDUs. Studies that attempted to identify risk factors of HCV/HIV co-infection among IDUs were either limited to male IDUs [17] or young IDUs (≤29 years of age) [15] or underestimated the effects of co-infection because, instead of seronegative cases, they enrolled mono-infected cases [16], or both mono-infected and seronegative cases, as the reference group [15,17]. Moreover, findings on the association between syringe sharing and HCV/HIV co-infection were not consistent [15-17]. Investigations about factors associated with HCV and HIV mono-infection and HCV/HIV co-infection among IDUs are needed to control the epidemic. Thus, this study aimed to measure the prevalence, incidence, and correlates of HCV/HIV co-infection and HCV mono-infection, as compared with seronegativity, among IDUs at a large methadone maintenance treatment program (MMTP) in Taipei, Taiwan in 2006–2010.

## Methods

### Study setting and population

In this cross-sectional study we reviewed the enrollment records for all clients at the MMTP of the Kun-Ming branch of Taipei City Hospital (TCH) between 2006 and 2010. TCH began their MMTP in December 2006 and voluntarily enrolled Taiwanese drug users with a history of heroin addiction by any route [14]. TCH serves approximately 90.6% of all MMTP clients in Taipei [14]. Every day, MMTP clients are required to attend methadone stands at hospitals to receive free therapy with methadone under direct supervision. They are also required to visit methadone clinics for counseling every month. Those who do not attend methadone stands for 2 weeks or longer are removed from the therapy list. If they seek re-enrollment, they are required to pay the equivalent of US$100 for biological testing. At TCH, the retention rate among MMTP clients was 60.7% and 43.4% at 6 and 12 months after enrollment, respectively [14]. All MMTP participants who had injected drugs in the 6 months before MMTP enrollment between 2006 and 2010 were included in this study. All MMTP clients who used only inhaled drugs were excluded from the analysis. This study was approved by the Institutional Review Board of TCH. All enrollees were anonymous.

### Data collection

At the time of enrollment, each MMTP participant completed a face-to-face interview conducted by trained case managers using a standardized questionnaire. The information collected included sociodemographic characteristics (i.e., age, gender, marital status, education, employment status, principal source of income in the 6 months before MMTP enrollment, living situation, number of MMTP enrollments, and history and frequency of incarceration) and substance use histories (i.e., age at first drug use, types of drugs used, syringe sharing in the 6 months before MMTP enrollment, types of drugs used in the 6 months before MMTP enrollment, and any alcohol use in the 6 months before MMTP enrollment). Principal source of income was classified as regular employment, social welfare or family, or other, including savings and friends. Living situation included living with family, living with friends, and living alone. The types of drugs used included heroin, amphetamine, marijuana, and others. For re-enrollees, data from their most recent interviews were used in the analysis. The existing dataset contained an identifier to indicate whether an individual was a re-enrollee. This identifier was coded by TCH prior to the data release and not by the researchers.

### Biological testing

At enrollment, MMTP clients are required to undergo serologic testing for HCV and HIV antibodies and urine drug screening. HCV and HIV antibody testing was performed by Kun-Ming Laboratory (Taipei, Taiwan). HCV antibody (anti-HCV) testing was done with a chemiluminescence immunoassay (Architect i 2000 version 3.0, Abbott Diagnostics) [18]. HIV testing was done with an enzyme-linked immunosorbent assay (Genscreen HIV1/2 version 2) followed by Western blot confirmation of reactive samples according to standard protocols [19]. Urine drug screening consisted of a competitive immunochromatography test (Firstep Bioresearch, Inc.) [20]. MMTP clients were required to visit MMTP clinics 1 week after enrollment to receive the above test results and associated counseling as well as referrals for clinical care if needed. In Taiwan, the government, together with the national health insurance program, covers HCV and HIV treatment. Re-enrolled participants underwent repeat HCV and HIV testing if they were negative for HCV or HIV at the preceding enrollment.

### Statistical analysis

Prevalences of HCV/HIV co-infection and HCV and HIV mono-infection were based on the most recent MMTP enrollment data for the study participants. HCV and HIV incidences were estimated from re-enrolled participants who were HCV- or HIV-negative at the preceding MMTP enrollment. Person-years of follow-up was used to estimate incidence rate and was calculated from the baseline to the midpoint between the last negative and first positive test results.

Study participants were divided into three groups: HCV/HIV co-infected, HCV mono-infected, and seronegative (i.e., negative for both HCV and HIV). Since the HCV and HIV co-infection is not uncommon in IDUs and these two infections shared the similar route of transmission, this study used HCV/HIV seronegativity as the reference group to determine the factors associated with HCV mono-infection and HCV/HIV co-infection, respectively.

In bivariate analysis, variables were analyzed using means or medians for continuous measures and frequencies and percentages for categorical variables. The chi-square test was used to assess bivariate associations of selected factors with HCV/HIV co-infection and HCV and HIV mono-infection. All variables found to be statistically significant (*P* < 0.10) in bivariate analysis were considered for inclusion in multivariable analysis [21]. Backward stepwise multinomial regression analysis was used because the dependent variable had more than two discrete outcomes. This yielded a final model that included factors with a *P* value < 0.05. Odds ratios (ORs) and adjusted odds ratios (AORs) with 95% confidence intervals (CIs) are reported in order to show the strength and direction of associations. Analyses were done with the SPSS version 19.0 statistical software package (SPSS, Chicago IL, USA).

## Results

### Description of the study population

A total of 1,447 IDUs participated in the TCH MMTP from December 2006 through the end of 2010; 13.1% (190) were HCV/HIV co-infected, 78.0% (1128) were HCV mono-infected, 0.4% (4) were HIV mono-infected, and 8.6% (125) were seronegative (Table 1). The 4 HIV mono-infected cases were excluded from subsequent analysis because of the small sample size. Among the remaining 1443 IDUs, the annual numbers of participants were 3 (0.2%) in 2006, 264 (18.3%) in 2007, 400 (27.7%) in 2008, 445 (30.8%) in 2009, and 331 (22.9%) in 2010. The mean age of the clients was 41 years (standard deviation, sd: 10); 86% were male; and the mean years of injecting drug use was 14.3 (sd: 9.7). 294 (20.4%) of the 1,443 study subjects were admitted to the TCH MMTP twice or more, including 64 HCV/HIV co-infections (33.7%, 64/190), 217 HCV mono-infections (19.2%, 217/1128), and 13 seronegativities (10.4%, 13/125) (Chi-square test for p value <0.001).

**Table 1:** Sociodemographic and substance use characteristics of participants by serologic status

	**Seronegative (n=125)**	**HCV and HIV co-infection (n=190)**		**HCV-monoinfected (n=1128)**	
**Factors**	**n(%)**	**n(%)**	**OR(95%CI)**	**n(%)**	**OR(95%CI)**
Sociodemographics					
Age (years), median (IQR)	35 (30–45)	40 (32–47)	1.03 (1.004-1.05)	40 (33–48)	1.04 (1.02-1.06)
Male gender	101 (80.8)	158 (83.2)	1.17 (0.65-2.11)	982 (87.1)	1.60 (0.99-2.58)
Year admitted, 2009-2010	51 (40.8)	111 (58.4)	2.01 (1.27-3.18)	614 (54.4)	1.72 (1.18-2.50)
Married status	25 (20.0)	40 (21.1)	1.07 (0.61-1.87)	247 (21.9)	1.12 (0.71-1.78)
Education level≦Primary school	10 (8.0)	30 (15.8)	2.16 (1.10-4.59)	194 (17.2)	2.39 (1.23-4.64)
Unemployment	67 (53.6)	118 (62.1)	1.42 (0.90-2.24)	652 (57.8)	1.19 (0.82-1.72)
Source of most income^a^					
Regular employment	77 (61.6)	103 (54.2)	1	689 (61.1)	1
Social welfare/Family	36 (28.8)	70 (36.8)	1.45 (0.88-2.39)	363 (32.2)	1.23 (0.74-1.71)
Others	12 (9.6)	17 (8.9)	1.06 (0.48-2.35)	76 (6.7)	0.71 (0.37-1.36)
Living situation					
Live with family	95 (76.0)	134 (70.5)	1	860 (76.2)	1
Live with friends	20 (16.0)	31 (16.3)	1.10 (0.59-2.04)	112 (9.9)	0.62 (0.37-1.04)
Live alone^b^	10 (8.0)	25 (13.2)	1.77 (0.81-3.86)	156 (13.8)	1.72 (0.88-3.38)
Lifetime MMTP admissions, mean (range)	1.14 (1–4)	1.41 (1–4)	2.63 (1.57-4.39)	1.22 (1–4)	1.49 (0.94-2.37)
Lifetime incarcerations, median (IQR)	1 (0–2)	4 (2–5)	1.92 (1.64-2.25)	3 (2–5)	1.82 (1.58-2.10)
Substance use histories					
Age at first injection drug use ≤20	31 (24.8)	66 (34.7)	1.61 (0.98-2.67)	315 (27.9)	1.18 (0.77-1.80)
Length of drug injection (years), mean (range)	9.28 (1–34)	14.56 (1–49)	1.10 (1.06-1.14)	14.76 (1–64)	1.08 (1.05-1.11)
Shared syringes^a^	11 (8.8)	135 (71.1)	25.44 (12.71-50.90)	161 (14.3)	1.73 (0.91-3.28)
Smoking amphetamine^a^	75 (60.0)	106 (55.8)	0.84 (0.53-1.33)	514 (45.6)	0.56 (0.38-0.81)
Smoking Marijuana^a^	119 (95.2)	184 (96.8)	1.55 (0.49-4.91)	1081 (95.8)	1.16 (0.49-2.77)
Used cocaine^a^	5 (4.0)	5 (2.6)	0.65 (0.18-2.29)	43 (3.8)	0.95 (0.37-2.45)
Swallowed sedative drugs^a^	17 (13.6)	28 (14.7)	1.10 (0.57-2.10)	145 (12.9)	0.94 (0.55-1.61)
Any alcohol use^a^	19 (15.2)	20 (10.5)	0.66 (0.34-1.29)	114 (10.1)	0.63 (0.37-1.06)
Urine morphine testing^c^	113 (90.4)	167 (87.9)	0.77 (0.37-1.61)	1033 (91.6)	1.16 (0.61-2.17)
Urine amphetamine testing^c^	72 (57.6)	48 (25.3)	0.46 (0.28-0.74)	829 (73.5)	0.49 (0.34-0.72)

HCV, hepatitis C virus. HIV, human immunodeficiency virus. OR, odds ratio. CI, confidence interval. IQR, interquartile range. MMTP, methadone maintenance treatment program.

^a^ During the 6 months before enrollment in the MMTP.

^b^ Including one homeless injection drug user.

^c^ At the time of enrollment in the MMTP.

### Covariates of infection: univariate and multinomial regression analyses

As shown in Table 1, the chi-square test revealed that the factors significantly positively associated with HCV/HIV co-infection included age, enrollment during 2009–2010, primary-school education or less, greater number of MMTP enrollments, higher frequency of incarceration, longer duration of injecting drug use, and syringe sharing in the 6 months before MMTP enrollment. Additionally, amphetamine positivity on urine testing at enrollment was significantly inversely associated with co-infection. Variables significantly positively associated with HCV mono-infection included age, enrollment during 2009–2010, primary-school education or less, higher frequency of incarceration, and longer duration of injecting drug use. Variables significantly inversely associated with HCV mono-infection included smoking amphetamine in the 6 months before MMTP enrollment and amphetamine positivity on urine testing at enrollment.

In backward-stepwise multinomial regression, after controlling for study subjects’ sociodemographics and substance use histories, the odds of HIV infection was 27 times higher (95% CI 13.30–57.76) among IDUs with syringe sharing in the 6 months before MMTP enrollment than among other IDUs without syringe sharing (Table 2). Also HCV/HIV co-infection was significantly positively associated with number of MMTP enrollments (AOR=2.28, 95% CI 1.33–3.90), and number of times incarcerated (AOR=2.01, 95% CI 1.71–2.37) and significantly inversely associated with smoking amphetamine in the 6 months before MMTP enrollment (AOR=0.44, 95% CI 0.25–0.76; Table 2). Higher number of times incarcerated increased the risk of HCV mono-infection by 1.77 times (95% CI 1.52–2.06). Additionally, HCV mono-infection was significantly positively associated with duration of injecting drug use (AOR=1.04, 95% CI 1.01–1.07) and was significantly inversely associated with smoking amphetamine in the 6 months before MMTP enrollment (AOR=0.49, 95% CI 0.32–0.75) and amphetamine positivity on urine testing at MMTP enrollment (AOR=0.61, 95% CI 0.40–0.93).

**Table 2:** **Demographic and substance use variables associated with HCV**/**HIV co**-**infection and HCV mono**-**infection in backward stepwise multinomial regression analysis**

	**AOR**	**95%CI**
HCV and HIV co-infection		
Shared syringes^a^	27.72	13.30-57.76
Number of MMTP enrollments (per time increase)	2.28	1.33-3.90
Number of times incarcerated (per time increase)	2.01	1.71-2.37
Smoking amphetamine^a^	0.44	0.25-0.76
HCV mono-infection		
Number of times incarcerated (per time increase)	1.77	1.52-2.06
Length of drug injection (per year increase)	1.04	1.01-1.07
Smoking amphetamine^a^	0.49	0.32-0.75
Positive amphetamine urine drug screen^b^	0.61	0.40-0.93

Reference is HCV/HIV-seronegative cases.

*AOR,* adjusted odds ratio. *CI,* confidence interval. *HCV,* hepatitis C virus. *HIV,* human immunodeficiency virus. *MMTP,* methadone maintenance treatment program.

^a^ During the 6 months before enrollment in the MMTP.

^b^ At the time of enrollment in the MMTP.

### HCV and HIV incidences among re-enrollers

During the study period, 14 HCV seroconversions were detected among the 24 re-enrolled IDUs who were negative for HCV at the preceding enrollment. This yielded a total of 30.9 person-years at risk (PYAR) and a crude incidence rate of HCV mono-infection of 45.25 (95% CI 24.74–75.92)/100 PYAR. 3 (21.4%) of the 14 HCV seroconverters and 2 (20%) of the 10 still HCV negativities had syringe sharing in the last 6 months prior to reentry into the MMTP. Additionally, 2 HIV seroconversions were detected among the 236 re-enrolled IDUs who were HIV-negative at the preceding enrollment, which yielded a total of 378.9 PYAR and a crude incidence rate of 0.53 (95% CI 0.06-1.91)/100 PYAR. The 2 HIV seroconverters denied sharing syringes in the last 6 months prior to reentry into MMTP. Furthermore, no HCV/HIV co-infection case was detected among the 23 re-enrolled IDUs who were negative for both HCV and HIV at the preceding enrollment. The small number of seroconversions precluded further analysis of risk factors for incident HCV and HIV infections.

## Discussion

According to the above analysis, we found that the prevalences of HCV/HIV co-infection and HCV mono-infection were high among IDUs enrolled in the Taipei City Hospital MMTP between 2006 and 2010. After controlling for other characteristics, syringe sharing in the 6 months before MMTP enrollment was significantly positively associated with HCV/HIV co-infection but not with HCV mono-infection. Additionally, greater frequency of incarceration was significantly positively associated with HCV/HIV co-infection and HCV mono-infection, whereas smoking amphetamine in the 6 months before MMTP enrollment was inversely associated with HCV/HIV co-infection and HCV mono-infection.

Although many studies have investigated the seroepidemiology of HCV infection among IDUs in Taiwan [9-11,22-24], none distinguished between HCV mono-infection and HCV infection, and only one evaluated risk factors of HCV infection [11]. Chang et al. found that younger age and longer duration of injecting drug use were independently associated with HCV infection among Taiwanese IDUs; however, because that study used a combination of HIV mono-infected and seronegative cases as the reference group, it may have underestimated the effects of HCV infection [11].

A literature review showed that the prevalence of HCV/HIV co-infection among TCH IDUs (13.1%) was not much different from that among whole IDUs population in China in 2007 (12.7%) [25] but higher than among incarcerated IDUs in the United States between 2003 and 2004 (4.4%) [26] and lower than among incarcerated IDUs in Iran in 2006 (24%) [17]. Although few studies evaluated the prevalence of HCV mono-infection among IDUs, overall HCV prevalence (91.1%) was higher in our study than in prior reports in Taiwan. For example, HCV prevalence was 59.5–89.6% among incarcerated IDUs between 1997 and 2005 [9-11] and 89.2% among IDUs at the MMTPs in four counties and cities in 2008 [12]. The present overall HCV prevalence was also higher than that reported in most other Asian countries, e.g., 47.8% in India between 2004 and 2006 [27], 60% in China in 2007 [28], and 46% in Vietnam between 2005 and 2006 [29]. This extremely high prevalence of HCV among IDUs in Taipei must be addressed by effective HCV prevention and better access to HCV therapy.

Syringe sharing in the 6 months before MMTP enrollment was strongly associated with HCV/HIV co-infection in this study. This finding may reflect that injection practices were the major transmission route of HCV and HIV infection among IDUs [3,5]. Because co-infection with HCV and HIV in IDUs has become a rapidly emerging global public health problem [7,8], interventions including syringe exchange services and drugs substitution treatments are imperative to prevent IDUs from HCV and HIV infections [30].

We found that greater frequency of incarceration was significantly associated with HCV/HIV co-infection and HCV mono-infection, which suggests that IDUs with a history of incarceration were more likely to have risk behaviors related to blood-borne infection [31]. Future studies should attempt to identify the risk behaviors that result in such infections.

Smoking amphetamine in the 6 months before MMTP enrollment was significantly inversely associated with HCV/HIV co-infection and HCV mono-infection. Amphetamine and opiates (e.g., heroin and morphine) have contrasting behavioral effects [32]. Thus, IDUs who smoked amphetamine would be less likely to inject opiates and consequently would have fewer injection risk behaviors and a lower risk of HCV/HIV co-infection and HCV mono-infection. This finding supports the harm reduction policy of providing clean syringes and methadone substitution treatment for IDUs.

HCV incidence among re-enrolled IDUs who were HCV-negative at the preceding MMTP enrollment was 45.25/100 PYAR in this study. HIV incidence among re-enrolled IDUs who were HIV-negative at the preceding MMTP enrollment was 0.53/100 PYAR, which is 74 times higher than that among the general population [14]. High incidences of HCV and HIV among IDUs who discontinued methadone treatment suggest that HCV and HIV transmission among IDUs in Taipei remains a serious concern. Prior studies showed that interruptions to methadone treatment were associated with increased prevalence of injecting drugs and sharing syringes [33], which could raise risks of HCV and HIV infection. Such findings support recommendations to provide HCV and HIV risk reduction counseling, including messages on safe injecting, to IDUs at MMTP enrollment.

Caution is required in interpreting the results of this study. First, we analyzed secondary data; information on the sexual behaviors and occupation of the study participants was not available. However, the sexual behavior of the participants is not likely to have confounded the main findings on risk factors associated with HCV mono-infection and HCV/HIV co-infection because HCV transmission via risky sexual behavior is not common [34]. Second, the use of cross-sectional data, without information on the temporal sequence between risk behaviors and infection of individual IDUs, precludes causal inference. Third, it is difficult to obtain a truly representative sample of a population of community-dwelling IDUs. It has been estimated that MMTP participants accounted for fewer than 20% of reported heroin-addicted drug users in Taipei in 2010 [14]. Thus, the generalizability of the present findings might be limited to IDUs at the Taipei MMTP.

## Conclusions

This appears to be the first study to estimate seroprevalence and seroincidence of HCV/HIV co-infection and HCV mono-infection among IDUs at an MMTP in Taipei. IDUs at a Taipei MMTP had a very high prevalence of HCV/HIV co-infection and HCV mono-infection. Interventions such as expansion of syringe exchange programs and education on HCV/HIV prevention must be implemented for this high-risk group of drug users.

## Competing interests

The authors declare that they have no competing interests.

## Authors’ contributions

YFY and CYD conceived and designed the study; YFY, CYD, MYY, LWS, LHL, PC, and XRJ analyzed and interpreted the data. All authors contributed to drafting the manuscript and revising it critically for important intellectual content, and read and approved the final version.

## Pre-publication history

The pre-publication history for this paper can be accessed here:

http://www.biomedcentral.com/1471-2458/12/1066/prepub
